# How University students in Bangladesh engage with ChatGPT: A qualitative study

**DOI:** 10.1371/journal.pone.0333089

**Published:** 2025-09-23

**Authors:** Mir Hasib, Md. Shariful Islam

**Affiliations:** 1 College of Communication & Information Sciences, University of Alabama, Tuscaloosa, Alabama, United States of America; 2 Department of Mass Communication and Journalism, Social Science School, Khulna University, Khulna, Bangladesh; National Taiwan University, TAIWAN

## Abstract

This study examined university students’ perceptions and practices of using ChatGPT through a qualitative approach, employing semi-structured, in-depth interviews with 20 Khulna University students. Using thematic analysis, the research identified key themes, including both academic and non-academic motivations, with applications ranging from assignments and research to entertainment. Despite limitations such as false references and predetermined feedback, students found ChatGPT efficient and useful both inside and outside the classroom. However, concerns about academic integrity arose, as some students devised creative ways to bypass existing plagiarism detection systems. Ethical considerations emerged regarding responsible AI use, underscoring the need for clear guidelines and training to ensure proper referencing and the development of critical thinking skills. Future research should focus on continuous evaluation and the implementation of support services to encourage the responsible integration of ChatGPT in university settings.

## Introduction

Artificial Intelligence (AI) chatbots have seen a meteoric rise in popularity over the past few years since they provide users with a novel and time-saving method of communication [1]. AI Chatbots are becoming increasingly sophisticated as a result of developments in artificial intelligence and natural language processing. They are currently being used in a variety of applications, including customer service, healthcare, and education [2].

ChatGPT-3.5, an advanced language model developed by OpenAI, has gained significant attention and popularity in recent years [3]. It is a generative AI that can generate text, images, code, and more. Through its extensive training on social media, websites, articles, datasets, books, and other internet texts, it can answer questions and help humans with tasks [4]. It has reached an estimated 100 million active users per month. However, the exact number of active users is unknown. Brandl [5] estimated the number to be approximately 100 million, if 10% of their website visitors sign up for the free service. ChatGPT was trained on a 300-billion-word dataset, which is approximately 570GB [6]. ChatGPT-3.5 was launched as a prototype on November 30, 2022, and was praised for its detailed and articulate responses across many fields [5]. ChatGPT-4, an updated version, launched in March 2023. This version is only available to ChatGPT Plus users or API developers [6].

The emergence of ChatGPT represents a significant advancement in natural language processing and AI [4]. With its impressive ability to generate coherent and contextually relevant responses, ChatGPT has found utility in various domains. It has emerged as a transformative tool in education, offering personalized learning experiences, efficient academic support, and opportunities for skill development. Its integration into educational settings has garnered significant attention due to its ability to streamline complex academic processes and foster interactive learning environments.

ChatGPT facilitates tailored learning experiences by adapting to individual student needs. It provides personalized feedback, automates assessments, and supports independent learning, making it an essential tool for modern education systems. For example, in programming education, ChatGPT has been shown to enhance computational thinking and algorithmic skills while improving creativity and problem-solving abilities. Similarly, in nursing education, ChatGPT has significantly boosted academic performance and research capabilities [7,8].

ChatGPT offers quick responses to queries, saving time for students and faculty alike. Its ability to synthesize information into concise formats reduces the need for extensive research across multiple sources. Additionally, it aids in language skill development by providing grammar corrections and vocabulary suggestions. However, concerns about accuracy persist, as ChatGPT sometimes generates false references or inconsistent outputs [9,10].

Recent studies highlight ChatGPT’s capacity to motivate students by fostering active learning processes. It improves engagement through interactive dialogues and tailored coaching, which are particularly beneficial in disciplines like English language learning [8,11]. Moreover, its use in creating collaborative environments has been linked to enhanced psychological well-being among students [9].

Despite its advantages, ethical challenges such as plagiarism and over-reliance on AI tools remain prevalent. Students often employ ChatGPT to complete assignments or exams without proper attribution, raising concerns about academic integrity. Educators emphasize the need for guidelines to ensure responsible usage while promoting critical thinking skills [12,13].

University students, in particular, have shown a keen interest in using ChatGPT for a range of academic and personal purposes. It can understand and respond to natural language queries, making it an ideal tool for education [14]. The use of ChatGPT in education can have a significant impact on student learning, as it can provide personalized assistance to students and help them overcome their learning challenges [15]. University students use ChatGPT for a variety of reasons. While some use it for its ability to personalize content and automate tedious tasks, others have been found using it to cheat in classes [16]. This has led to discussions among faculty and administrators about the ethical use of ChatGPT in educational settings [17].

While existing studies have explored the applications of ChatGPT in education, there is limited research on the motivations driving student usage. The Uses and Gratifications Theory (UGT) provides a framework for understanding these motivations but remains underutilized in this context. Students use ChatGPT for cognitive gratifications (information-seeking), affective gratifications (emotional satisfaction), personal integrative gratifications (self-awareness), and social integrative gratifications (peer interaction). However, the extent to which these motivations influence learning outcomes or ethical behavior remains unexplored. This study aims to explore the factors of using ChatGPT that facilitate academic assistance in higher education and to explore the ethical implications of ChatGPT usage in academic settings.

### Significance of the study

Studying ChatGPT usage among Bangladeshi students contributes meaningful insights into the broader discourse on AI integration in education. Investigating how Bangladeshi students perceive and utilize ChatGPT helps identify specific challenges and opportunities unique to developing countries with similar socioeconomic conditions. Insights from this study can guide policymakers in formulating regulations for ethical AI use while promoting accessibility and inclusivity in higher education systems [13]. By focusing on student motivations and perceptions, this study bridges gaps in existing literature regarding the psychological and behavioral factors influencing AI adoption in education [18,19]. In conclusion, exploring the impact of ChatGPT on Bangladeshi students provides valuable contributions to understanding AI’s role in transforming education globally while addressing localized challenges. This research underscores the importance of balancing technological benefits with ethical considerations to ensure sustainable integration into academic practices.

## Literature review

The integration of AI tools into educational environments has significantly accelerated since the advent of advanced AI models like ChatGPT-4, released in March 2023. The use of AI tools such as ChatGPT and intelligent tutoring systems has revolutionized personalized learning by customizing educational content according to individual student needs. These tools provide real-time feedback, adaptive learning paths, and tailored resources, facilitating self-paced learning and addressing specific academic challenges effectively. For instance, OpenAI’s GPT-4o effectively guides students through complex problems without directly providing answers, thereby encouraging critical thinking and learner autonomy [20,21].

Beyond enhancing student learning experiences, AI technology has streamlined numerous administrative tasks. Educators increasingly leverage AI for automated grading, lesson planning, and assessment creation, freeing valuable time for interactive teaching and mentorship roles [22,23]. A notable development is ChatGPT Edu, launched in 2024, specifically designed to assist universities in grading and personalized tutoring, demonstrating AI’s growing role in educational management [20].

AI tools have notably expanded inclusivity in education, employing technologies such as text-to-speech, visual recognition, and adaptive interfaces, thereby addressing the needs of neurodiverse students and those with disabilities. These innovations help reduce educational disparities, ensuring broader access to quality education [24]. Moreover, AI-driven platforms such as Canva Magic Write and Curipod are facilitating student collaboration and creativity, assisting them in generating high-quality instructional materials and assignments efficiently [25].

Recent literature provides an interdisciplinary perspective on AI’s transformative impacts, advocating a human-centered approach for its integration into education [26]. Research highlights that ChatGPT’s context-aware responses significantly enhance student engagement and reduce instructional burdens, underlining the importance of balanced integration, digital literacy, and authentic assessment practices [27]. Meta-analyses further confirm that AI tools significantly boost cognitive, behavioral, and emotional engagement, demonstrating their role as tutors, assistants, and collaborators [28]. A synthesis of 21 case studies employing pedagogical models (TPACK, SAMR, Laurillard) further underscores how generative AI supports personalized feedback, collaborative writing, and critical thinking skills development [29]. All these connects ChatGPT use to emotional and behavioral engagement, suggesting deeper psychological and motivational dynamics behind usage patterns [28].

Recent survey data reflects a significant rise in AI adoption among students, with the Digital Education Council’s 2024 survey reporting that 86% of higher education students actively use AI, predominantly ChatGPT, for academic purposes such as summarizing texts, paraphrasing, and drafting essays. Despite this extensive adoption, approximately 58% of students still feel inadequately prepared for an AI-driven workforce, highlighting a critical gap in AI literacy and underscoring the urgent need for comprehensive AI training within educational curricula [30,31].

The ethical implications surrounding AI usage in academia are pronounced, particularly concerning academic integrity and plagiarism. A study by Welding revealed that while many students view AI tool use as cheating, a significant 43% still employ these tools for assignments and examinations [32]. Universities such as Flinders and Adelaide have adopted policies permitting regulated AI usage in academic work, conditional on adherence to clear guidelines, stressing critical thinking verification exercises alongside AI-generated outputs [33]. The University of Queensland advises students to verify their course requirements before incorporating AI in assessments to emphasize the importance of critical thinking [34].

Interactive textbooks and chatbot applications have become widespread, significantly contributing to student engagement by simulating human-like dialogues that promote language acquisition and communication skills [35–37]. Nonetheless, challenges remain, particularly regarding the optimal integration of chatbots into education without negatively impacting the learning experience [37,38].

Personalized learning facilitated by AI addresses individual educational requirements effectively, yet limitations such as the rigidity of AI-generated tasks and a scarcity of appropriate resources hinder full realization of its potential [39,40]. Educators integrating AI technologies into classroom management often encounter challenges due to limited understanding of these technologies, resulting in feelings of loss of control over teaching strategies [41–43].

AI-driven systems also benefit educators’ professional development through real-time data analysis, offering personalized feedback that improves teaching methodologies. Nevertheless, limitations remain concerning the breadth of AI-generated recommendations and the compatibility of predictive models with diverse student data sets [44–48]. AI-powered assessment tools significantly enhance grading accuracy and efficiency, though their application remains predominantly confined to specific areas such as language acquisition [49–51].

However, AI’s ease in content generation has amplified concerns regarding plagiarism, ethical misuse, and biased or inaccurate information outputs, necessitating robust guidelines and strategies for responsible AI integration into educational practice [30,52]. Studies from Southern Asian contexts indicate a correlation between AI usage and decreased academic integrity, driven by behavioral changes such as procrastination and diminished classroom engagement [53]. Experimental research further cautions that reliance on AI tools like ChatGPT can significantly diminish creativity and originality, especially within creative disciplines, underscoring the need for cautious integration and policy regulation [54].

To address these multifaceted challenges, institutions and policymakers must actively promote AI literacy and ethical standards. Initiatives like UNESCO’s AI competency framework and legislative efforts such as California’s inclusion of AI literacy in K-12 curricula underscore the importance of responsible AI use [20,54]. Furthermore, recent advancements in multimodal AI models (e.g., GPT-4o) present new educational opportunities through enriched audio, video, and image capabilities, necessitating careful implementation to ensure equity and accessibility [21].

While AI has substantially reshaped educational practices through enhanced personalization, administrative efficiency, and creative support, it concurrently poses significant ethical and practical challenges. Addressing these issues requires comprehensive AI literacy training, equitable access strategies, robust ethical frameworks, and ongoing research into AI’s long-term impacts on education.

## Theoretical framework

The Uses and Gratification Theory (UGT) is a communication theory that was developed in the early 1940s by Katz and Blumler [55]. It seeks to explain why people use various forms of media and what gratifications they seek from those forms of media [56]. Following similar studies [57–60], this theory can be adapted to explore the motivations for using ChatGPT among university students.

Individuals actively choose and use various forms of media to satisfy their various needs and desires, as stated by UGT. The theory identifies four categories of gratifications that individuals seek from media: cognitive, affective, personal integrative, and social integrative [56]. Cognitive gratifications refer to the need for information, knowledge, and understanding [59]. Affective gratifications refer to the need for emotional experiences, such as pleasure, excitement, and relaxation [58]. Personal integrative gratifications refer to the need for self-awareness, self-esteem, and personal identity [57]. Social integrative gratifications refer to the need for social interaction, companionship, and a sense of belonging [60].

Motivation can be defined as the driving force behind an individual’s behavior or action [61]. In this study, motivation refers to the gratifications that students seek from ChatGPT, such as cognitive, affective, personal, and social integrative gratifications. Inspired by Urbancová and Fajčíková [62] A student may be motivated to use ChatGPT to seek information and knowledge on a particular topic (cognitive gratification), emotional experiences, such as humor or empathy, from the chatbot’s responses (affective gratification), self-awareness and personal identity through the chatbot’s responses (personal integrative gratification), or social interaction and companionship through the chatbot’s responses (social integrative gratification).

While UGT provides a robust framework for understanding media usage motivations, it has notable limitations when applied to the context of ChatGPT usage among university students. UGT primarily focuses on individual needs and gratifications, often neglecting broader social, cultural, and institutional factors that influence media consumption. For example, students’ use of ChatGPT may be shaped by academic pressures, institutional policies, or peer influence, which are not fully addressed by UGT. Although UGT explains why individuals use certain media, it struggles to predict how usage patterns evolve over time or in response to technological advancements. For instance, the rapid adoption of ChatGPT may be driven by trends rather than intrinsic motivations, which UGT does not adequately capture. UGT assumes that users are aware of their motivations, but unconscious or subconscious factors often play a role in media consumption.

Students may use ChatGPT impulsively or habitually without consciously seeking specific gratifications. The theory emphasizes the active role of users while overlooking the influence of media producers and the design of AI tools like ChatGPT. For example, ChatGPT’s user-friendly interface and its ability to generate instant responses may shape user behavior beyond their conscious choices. Research relying on self-reporting methods under UGT may lead to biased or incomplete data regarding motivations. Students might underreport unethical uses (e.g., plagiarism) or overstate productive uses due to social desirability bias [63].

In the context of this study, university students may use the ChatGPT to satisfy their cognitive gratifications by seeking information and knowledge on various topics. They may also use the ChatGPT to satisfy their affective gratifications by seeking emotional experiences, such as humor or empathy, from the chatbot’s responses. Moreover, students may use ChatGPT to satisfy their integrative gratifications by seeking self-awareness and personal identity through the chatbot’s responses. Finally, students may use ChatGPT to satisfy their social integrative gratifications by seeking social interaction and companionship through the chatbot’s responses.

The current study identifies four primary categories of gratifications: cognitive, affective, personal integrative, and social integrative. All of them derived from UGT and connects them to specific motivations for using ChatGPT.

For Cognitive Gratifications, students use ChatGPT primarily for seeking information and knowledge. For example, completing assignments by generating ideas or summarizing key points or preparing for exams by creating concise notes. UGT informs research questions about how students perceive ChatGPT as a tool for enhancing academic productivity.

For Affective Gratifications, emotional experiences such as humor or relaxation are sought by some students engaging with ChatGPT informally, such as asking humorous questions or seeking entertainment during leisure time or using ChatGPT responses to alleviate stress during tight deadlines UGT can help to explore how emotional satisfaction influences the frequency and type of ChatGPT usage.

For Personal Integrative Gratifications, some students seek self-awareness or personal identity enhancement through ChatGPT, like career planning advice and decision-making assistance (e.g., choosing TV shows or recipes). UGT helps frame questions about whether such usage fosters self-confidence or dependency on AI.

For Social Integrative Gratifications, social interaction and companionship are reflected in students’ discussions about ChatGPT with peers, such as sharing prompts and results within friend circles or recommending ChatGPT as a resource for academic tasks. UGT can help investigate how social dynamics shape attitudes toward AI tools. By integrating these dimensions into the study design, UGT enables a nuanced exploration of how diverse motivations drive the usage of AI tools like ChatGPT in educational settings.

Research Objectives and Questions:

As guided by the UGT and based on the literatures, this study attempts to explore university students’ perceptions and practices regarding the use of ChatGPT, identify the motivations behind its academic and non-academic applications, and examine the ethical implications and challenges related to academic integrity and responsible AI use. Therefore, the following research questions are proposed:

RQ1. How do university students perceive and practice the use of ChatGPT in academic and non-academic contexts?

RQ2. What ethical and pedagogical challenges emerge from its integration in higher education?

## Method

To answers these research questions, this study adopted a qualitative approach, specifically, an exploratory study inspired by a Gruzd [60] to collect data from university students who used ChatGPT to understand their motivations. Qualitative research is well-suited for examining individuals’ subjective experiences, attitudes, and motivations, allowing for in-depth exploration and rich data collection [64]. In this study, the primary method utilized is in-depth interviews and semi-structured questionnaire, which provide a comprehensive understanding of the participants’ perspectives and motivations related to ChatGPT usage.

For this reason, purposive sampling was used in this research instead of random sampling to get our data. It is a non-probability sampling technique, to select participants who met specific criteria relevant to the research objectives. Purposive sampling allowed the researchers to deliberately recruit participants who were regular students at Khulna University and had used ChatGPT. This approach was both methodologically appropriate and logistically convenient, as both researchers are affiliated with the same university. By targeting students with direct experience using ChatGPT, the researchers could gather data that directly addressed the study’s UGT framework.

20 semi-structured in-depth interviews were conducted as part of the exploratory study with students at both the undergraduate and graduate levels. The size of the sample was determined based on data saturation, which is the point at which the interviews no longer provide any new insights or information. Previous researchers found that data saturation in qualitative interviews often occurs within the first 12 interviews, and basic themes can emerge as early as six interviews. Previous research demonstrates that “9–17 interviews” are typically sufficient to reach saturation, especially for studies with a homogenous population and narrowly defined objectives [53]. This ensured that the samples of the current study was adequate for capturing a comprehensive range of perspectives without unnecessary redundancy.

Due to the researchers’ convenience, the participants in this study consist of only Khulna University students. There were two inclusion criteria: (1) the Participant must be a regular student at Khulna University, and (2) must have experience using ChatGPT at least 3 times. The purposive sampling technique was employed to ensure diversity in terms of age, sex, academic disciplines, and levels of study. Each member was selected from different disciplines, including humanities, social sciences, natural sciences, and professional fields like engineering and architecture. This ensured that the findings reflected a wide range of academic contexts and needs. Also, participants included both undergraduate and graduate students, ensuring representation across different stages of academic progression. This allowed the study to capture variations in ChatGPT usage based on academic maturity and workload, see Table 1. The participants’ ages ranged from 21 to 28 years old. The breakdown for sex was 63.64 percent males and 36.36 percent females. Participants were chosen to reflect diverse usage scenarios of ChatGPT, such as academic tasks (e.g., assignments, thesis writing), personal development (e.g., career planning), and leisure activities (e.g., entertainment). This enriched the dataset by incorporating varied motivations and experiences.

**Table 1:** Background Information of the Participants.

*Serial No*	*Pseudonym*	*Age*	*Sex*	*Program*	*Year*	*Discipline*	*Length of Interview (mins.)*
1	Ali	28	Male	Masters	First	MCJ^k^	00:53:00
2	Akter	25	Female	Masters	Second	MATH^q^	00:42:00
3	Begum	27	Female	Honors	First	ECON^h^	01:01:00
4	Biswas	22	Male	Honors	First	SOC^t^	01:02:00
5	Chakraborty	25	Male	Honors	Fourth	LAW^p^	00:41:00
6	Das	26	Female	Masters	Second	DS^m^	00:50:00
7	Dewan	24	Male	Honors	Third	STAT^u^	01:07:00
8	Ghosh	28	Female	Honors	Third	FMRT^o^	00:56:00
9	Hossain	21	Male	Honors	Second	SWE^n^	00:58:00
10	Islam	27	Male	Honors	Fourth	PHAM^r^	00:59:00
11	Karim	23	Male	Honors	Second	ARCH^c^	01:08:00
12	Khan	26	Male	Honors	Third	BA^e^	01:14:00
13	Khatun	22	Female	Honors	First	BAN^d^	00:35:00
14	Miah	25	Male	Honors	Third	PM^j^	01:05:00
15	Mondal	23	Female	Honors	Fourth	ENG^l^	00:44:00
16	Paul	28	Female	Masters	First	CSE^g^	01:06:00
17	Roy	28	Male	Honors	Fourth	AT^b^	00:47:00
18	Saha	25	Female	Masters	Second	CHEM^f^	01:00:00
19	Sarker	25	Male	Honors	First	EDU^i^	01:03:00
20	Sheikh	26	Male	Masters	First	ECE^a^	01:07:00

^a^Electronics and Communication Engineering (ECE), ^b^Agrotechnology (AT), ^c^Architecture (ARCH), ^d^Bangla (BAN), ^e^Business Administration (BA), ^f^Chemistry (CHEM), ^g^Computer Science and Engineering (CSE), ^h^Economics (ECON), ^i^Education (EDU), ^j^Printmaking (PM) ^k^Mass Communication and Journalism (MCJ), ^l^English (ENG), ^m^Development Studies (DS), ^n^Soil Water and Environment (SWE), ^o^Fisheries and Marine Resource Technology (FMRT), ^p^Law (LAW), ^q^Mathematics (MATH), ^r^Pharmacy (PHAM), ^t^Sociology (SOC), ^u^Statistics (STAT).

Bangladesh presents a unique context for studying ChatGPT’s impact due to its evolving educational landscape and challenges such as large classroom setups, resource shortages, and low student participation rates [12]. There are several factors justify focusing on Bangladeshi students. Bangladesh is undergoing rapid technological transformation across various sectors, including education. University students are increasingly adopting AI tools like ChatGPT to address resource gaps and enhance academic productivity [13]. The socioeconomic conditions in Bangladesh make cost-effective tools like ChatGPT highly attractive for students who may lack access to premium educational resources such as paid writing assistants or tutors [12]. Bangladeshi students often face linguistic barriers when accessing global educational materials. ChatGPT’s ability to provide translations and summaries tailored to local contexts makes it particularly valuable in this setting [12]. Large class sizes and limited teacher-student interaction hinder personalized learning in Bangladeshi universities. ChatGPT offers solutions by providing immediate feedback and interactive support that can mitigate these issues [13].

Following an analysis of the pertinent literature, a semi-structured interview questionnaire was developed, see Supporting Information (S1 File). This questionnaire consisted of two modules that were incompatible with one another, the first of which was concerned with collecting primary data from the participants and was centered on demographic information. The second module consists of questions about the participant’s understanding, experience, and concerns about ChatGPT. It focused on students’ cognitive, affective, personal, and social needs, derived from the U&G theory within the educational context.

### Ethics statement

This study was approved by the Ethical Clearance Committee, and Director of Research and Innovation Centre of Khulna University (Reference No. KUECC – 2023-11-72). Participants were fully informed about the purpose of the study, the nature of their involvement, the procedures involved, and any potential risks or benefits. They were given the opportunity to ask questions and were informed of their right to withdraw from the study at any time without any penalty. This study did not involve any participants under the age of 18. Each participant verbally gave consent for participation. The verbal consent process was approved by the ethics committee, and the details of these interactions were recorded in our field notes. Therefore, parental or guardian consent was not required. Participants were encouraged to conduct interviews in private spaces to avoid interruptions or inadvertent breaches of confidentiality during the session. No waiver of consent was requested or granted for this study. All participants provided consent prior to their participation. Participation in this study was entirely voluntary. No benefits, financial or otherwise, were offered or given to participants in exchange for their involvement. Personal identifiers such as names, email addresses, or other identifiable information were removed from transcripts and replaced with pseudonyms. This ensured that participants’ identities remained confidential throughout the research process. Audio recordings and transcripts were stored on password-protected cloud storage systems to prevent unauthorized access. Only researchers directly involved in the study had access to these files. Participants were informed about how their data would be used, including anonymization procedures and secure storage practices. This transparency fostered trust and compliance with ethical standards.

### Interview outline

The interviews with the participants were conducted in person, as well as using Zoom, WhatsApp, and Messenger Calls. The interviews were conducted in December 2023. The researcher transcribed the conversation from Bengali to English language by following the Intelligent Verbatim method [65]. This was accomplished by uploading each audio-recorded conversation to the website happyscribe.com [66]. Two bilingual researchers also ensured semantic accuracy and clarity in translation, and a back-translation check was performed to maintain quality. After that, the UGT was applied to conduct the data analysis [58].

The researcher used NVivo 12 to perform open coding (marking several categories that developed from participants’ words, which were then distilled into more specific categories and themes) and in vivo coding (noting participants’ words that are indicative of user language). Open coding involves marking several categories that developed from participants’ words. After that, axial coding was performed by the researcher by condensing open codes into relevant subcategories [67,68]. The last step in the coding process was selective coding, which consisted of reorganizing categories to discover the primary themes hidden within the data. Subcategories were reorganized into broader themes to identify overarching patterns in ChatGPT usage motivations. The data were compiled using thematic analysis, which made use of the themes that were discovered. Data from interviews were cross-verified with existing literature on AI tools in education to validate findings against broader trends. Two researchers independently coded a subset of transcripts to ensure consistency in theme identification. Discrepancies were resolved through discussion until consensus was reached.

### Inclusivity in global research

Additional information regarding the ethical, cultural, and scientific considerations specific to inclusivity in global research is included in the Supporting Information (S2 File).

## Results

The RQ1 asked how do university students perceive and practice the use of ChatGPT in academic and non-academic contexts? The interviewees answered the following about the following:

### Academic use

#### Facilitating assignment and presentation.

Interviewees reported that they used ChatGPT to complete their class assignments and presentations. They used it to enhance productivity and efficiency while meeting deadlines. Some instructed ChatGPT to rewrite the entire assignment, while others sought its assistance in generating ideas. In instances where time was limited for crafting presentation slides, they asked ChatGPT to summarize, analyze, and highlight significant points from their assigned subjects. Generally, they received satisfying results, yet few adjustments were necessary before final submission due to partial alignment with their specific requirements.

Khatun (a 22-year-old female honors student from the BAN discipline) shared her experience on how she used ChatGPT to improve her assignment quality in this way:

“I used ChatGPT for submitting an assignment under a course. I was struggling to make key points while writing the assignment. One of my seniors told me about ChatGPT in a gossip. I used it for the first time and it gave me six relevant points on the topic. After giving multiple prompts, ChatGPT gave me 20 points. This elevated my assignment quality.”

Similarly, Mondal (a 23-year-old female honors student from the ENG discipline) used ChatGPT to write a story assignment. She stated this way:

“I was assigned to write a short story following the style of an English novelist and poet named Charlotte Bronte. I know that my writing will not be as near good as Charlotte’s. Then I remembered, that I saw a YouTube video a few days back where ChatGPT can generate or change a story mimicking any famous author’s style. So, I thought why don’t I give it (ChatGPT) a short. I only gave the contexts of the story and it surprised me with an amazing story. I changed the characters and location names in the context of Bangladesh before submission. I was one of the top scores in that assignment.”

Chakraborty explained his exposure to ChatGPT through a presentation as follows:

“My course teacher told me to compare and critique European Labour Law and the Bangladesh Labour Act. It was a hectic task to read and critically analyze all the important rules and regulations. I had only one night left before the presentation day. So, I used ChatGPT and it took only half an hour to prepare the whole speech.”

### Alternative to traditional note-taking

Participants used ChatGPT to prepare for their examinations. They reported that ChatGPT swiftly delivers information on a given topic, typically encompassing about 600 words, in a matter of seconds. These data are comparatively well organized and well written, which is convenient for the students to memorize and understand in a short period. Consequently, these students tend to forgo the practice of note-taking during lectures, as they found the convenience of having ChatGPT readily available on their devices to be a dependable alternative.

In this regard, Khan (a 26-year-old male honors student from the BA discipline) asserted how he used ChatGPT to create the notes for class tests:

“I prefer to study summarized topics before exams. Though I was shy, I had to go back and forth with my friends to collect notes for the exam as I don’t take notes during lectures. Now, using ChatGPT I can easily create my notes within a minute for class tests.”

### Replacing competing tools as a writing assistant

Even though there are plenty of applications like Grammarly and Quillbot, interviewee used ChatGPT as their writing assistant. They used it for a range of writing-related tasks, including grammar checker, paraphraser, and word extender. Competing products like Grammarly and Quillbot are noted for having word limits and reserving some features exclusively for premium users. The cost associated with accessing all features, especially for students, is considered expensive. As described by the interviewees, students are gradually accepting and adapting ChatGPT as a replacement for other writing assistant AI tools.

Biswas (a 22-year-old male honors student from the SOC discipline) stated how he used ChatGPT to improve his writing quality:

“After writing any essay I command ChatGPT to correct all the spelling and grammatical errors. I used to do this with Grammarly but it suggested multiple options, which consumes more time and effort. Sometimes, it recommended chain editing, which was frustrating. With ChatGPT, I can expand the word length by commanding simple prompts. On the other hand, there is no option for prompts in Quillbot. It has an expanding option as well as other features. However, both of these platforms have word limits. Only premium users can get access to all the features and exceeding the limit, which is expensive for me.”

#### Research assistance.

Respondents stated that they have used ChatGPT in their thesis. However, their experience did not match their expectation. They faced inconsistency and reference issues. ChatGPT gave them false references, made-up results, pre-prepared data, and unwanted information. Even though, it helped them to generate ideas and design concepts. They did not lose their motivation, instead, they accepted ChatGPT as it is and improvised their dissertation.

Akhter (a 25-year-old female master’s student from the MATH discipline) explained her usage of ChatGPT in her dissertation in this way:

“I asked ChatGPT to write an introduction part for my thesis. It wrote a good intro but first, it gave me no reference. Then, I asked for references and it gave me five sources. Unfortunately, only one reference was authentic and others were made up. However, I could not relate the text result with the only traceable reference. I guess, it gave me a random source.”

Islam (a 27-year-old male honors student from the PHAM discipline) shared his story:

“ChatGPT somewhat helped me to write my thesis methodology. It gave me some basic ideas. Although I expected a descriptive methodology, it gave me some key points without any reference. Also, every time it showed the whole research design from Introduction to Conclusion, which I did not ask for.”

#### Shifting search engine.

Respondents used ChatGPT as a search engine like Google. They believe that this AI will soon take over Google. Because ChatGPT saves their time by providing straight answers to their questions. They do not require visiting multiple links and reading long texts to find their desired answer. ChatGPT powered search feature has also made them shift from Google to Bing browser.

Sheikh (a 26-year-old male master’s student from the ECE discipline) stated this way:

“Using Google requires some time to find an appropriate answer to my questions. I needed to visit one link to another and read through whole paragraphs. Every time there will be pop-up advertisements, which makes me angry. Now I use ChatGPT from the Bing browser. It has a default ChatGPT access option and it does not require to log in every time. Most importantly, I get the direct answers right away. If I need to go deeper, I can ask more questions or visit the mentioned sources.”

### Non-academic use

#### Efficiency in content creation.

Interviewees admitted that ChatGPT helps them to improve the quality of writing job applications, emails, and CVs. It generates content quickly, reducing the time and effort required for written communication. However, they had to make some edits as it somewhat failed to merge with the job description to applicants’ data. They also said that ChatGPT works better if it is used for reply emails as it gets to process the previous conversations. Interviewees used ChatGPT for content creation, particularly for social media. For instance, students manage their discipline sports pages. During the sports season, they had to post multiple updates about their teams. In every post, they needed to be energetic and motivated. Whenever they ran out of creative speech and enthusiastic jargon, they asked ChatGPT for it.

Ali (a 28-year-old male master’s student from the MCJ discipline) revealed his content creation technique. He stated the following:

“In cricket and volleyball tournaments, I posted score updates and match results to our Facebook page. Just to make it sound more professional and enthusiastic I used ChatGPT to generate the captions.”

Saha (a 25-year-old female master’s student from the CHEM discipline) expressed how she used ChatGPT in this regard:

“While applying for an internship job at a research lab I asked ChatGPT to write me a cover letter. I copied the job description from LinkedIn, pasted it to ChatGPT, and told it to write the letter. It took only 10 seconds to give the result. Although I had to make some changes according to my CV.

Roy (a 28-year-old male honors student from the AT discipline) told his emailing experience in this way:

“I am planning to go abroad for higher study purposes. Every day I had to email multiple professors from North America and Europe to see if they were looking for any students in their labs. It is tough to write and reply to 20-30 emails every day for different people following different writing styles. Here, ChatGPT helped me to generate a common email in a matter of seconds.”

Ghosh (a 28-year-old female honors student from the FMRT discipline) explained the process in this manner:

“First I copied a job description to ChatGPT and told it to read it. Then I copied my CV in the same thread and told ChatGPT to rewrite it following the job description above. Then ChatGPT added an objective to my CV, it changed some words with the synonyms taken from the job description. However, it added some experiences and skills that were not in my CV before. I had to customize my CV again on a doc file but ChatGPT did a pretty good job to polish it.”

#### AI-powered leisure.

One respondent, Miah (a 25-year-old male honors student from the PM discipline) used ChatGPT for fun. He asked random questions to ChatGPT just to pass the time or entertain himself. ChatGPT has also shown its humor by engaging with him.

Miah expressed his feelings in this manner:

“All of my friends in the circle use ChatGPT. We love to discuss it and recommend others to try it. Apart from doing assignments, I use ChatGPT to ask stupid questions such as Who is the GOAT? Messi, Barrister Sumon, or Kazi Salahuddin? I asked ChatGPT to tell me some good jokes. It gave me some lame answers. However, earlier when ChatGPT launched, I asked will you take over the human race. It replied to me that You Never Know! Now it gives me a sophisticated answer. I find it fascinating to talk with an AI.”

#### Decision-making assistance.

Participants experimented with ChatGPT by using it as their decision-making assistant. They asked ChatGPT to provide recommendations at various decision-making phases and were pleased with the outcomes.

Hossain (a 28-year-old male honors student from the SWE discipline) reported his experimental journey in this way:

“Oftentimes, I ask ChatGPT which TV series I should watch or which game I should play. One day, I told ChatGPT some ingredients names that I had at that moment in my kitchen. I asked ChatGPT to give me a quick recipe. It gave me the recipe for Fried Rice.”

The RQ2 examined what ethical and pedagogical challenges emerge from its integration in higher education? The analysis found the following results:

### Ethical concerns

Interviewees confessed that they used ChatGPT to write assignments by plagiarism. However, they are not concerned about their ability of critical think critically critically and the importance of academic integrity. They take multiple measures including manual paraphrasing and altering context, to evade arousing suspicion from teachers and evading detection by online plagiarism detectors. On the other hand, some participants acknowledged potential ethical concerns about using ChatGPT, particularly during exams. During open-book exams, assignments, and the preparation of presentation materials, students made use of ChatGPT. There appears to be a fine line between acknowledging ethical concerns and using tools like ChatGPT to enhance academic performance.

#### Ethical dilemmas in exams.

Das (a 26-year-old female master’s student from the DS discipline) rationalizes the use of ChatGPT in open-book exams by equating it to a legitimate digital resource:

“I and some of my batch mates used ChatGPT during an ongoing class test. It was an open-book exam. ChatGPT is also a resource like a book, kind of a digital version. So, I don’t think that we broke any rules. However, to avoid any kind of trouble we didn’t notify our course teacher.”

#### Plagiarism practices.

Karim (a 23-year-old male honors student from the ARCH discipline) revealed how he did it without being caught:

“I always use Quillbot or ChatGPT itself to paraphrase the results. Therefore, if someone tries to check the assignment they won’t find any plagiarism. Even, if they do check it, the similarity result will be near 0%. Because I change some basic information according to my context. Sometimes, I write a few lines to add examples or information I have taken from class lectures to make it more authentic.”

#### Concerns about academic integrity.

Similarly, Dewan (a 24-year-old male honors student from the STAT discipline) shared his experience of avoiding being detected differently.

“There is no practice of checking assignments using Turnitin or any other AI detector sites. Also, most of the time assignments were submitted in a printed version. So, it is too difficult to check them, unless the teacher becomes too suspicious and takes the hassle.”

Despite ethical concerns, students prioritized performance enhancement over critical thinking development or adherence to academic integrity.

## Discussion

This study reveal that students used ChatGPT for both academic and non-academic purposes (RQ1). Academically, it supported assignment writing, idea generation, presentation preparation, and even replaced traditional note-taking and writing tools like grammar checkers and paraphrasers. It also served as an alternative to search engines by offering direct answers without browsing multiple sources. Non-academic uses included writing job applications, emails, and social media content, as well as casual entertainment and decision-making support. However, ethical concerns emerged, as some students admitted to using ChatGPT for plagiarism in assignments and open-book exams, highlighting a blurred line between academic support and misconduct (RQ2).

The majority of the interviewees believed that ChatGPT is more efficient and effective than existing methods, this supports the study of Palasundram [36], Porter and Grippa [69], Niloy [70], and Belkina [29]. They have found that ChatGPT has been used to provide guidance and feedback. On the other hand, some of the students complained about how ChatGPT gives false references, made-up results require multiple prompts to get a near result, has login issues, and sometimes stops responding. Similarly, Holstein [71], Farrokhnia [27], and Vazquez-Cano [38] argued about the difficulties and limitations of human-AI conversation. They revealed that the feedback that was provided by these systems was pre-prepared and did not fulfill the requirements of every student. This study also found that several students admitted to using ChatGPT for assignments, presentations, and even open-book exams, raising serious concerns around plagiarism and academic dishonesty. Despite widespread perceptions of AI-assisted work as cheating, 43% of students still reported using such tools in academic tasks [32]. This highlights a growing tension between ethical standards and practical utility, as students prioritize efficiency over integrity.

Although Chew and Chua [37] found the time and reason to use chatbots to promote student learning and engagement unclearly, our study showed that it can be applied both in and off the classroom, even for daily life learning purposes. Students have used ChatGPT to build their daily schedules for studying or entertaining. Some previous studies [70,39,41,42,72–74] have shown that AI has been used to customize student learning assignments by teachers or university authorities. Recently, some universities permitted students to use ChatGPT with the declaration [33], also they need to provide references as well as put their argument and exercise critical thinking [34]. Some participants stated that they took the initiative and openly used ChatGPT during presentations and class tests. Though all the interviewees used ChatGPT to write assignments and some of them used it to conduct their research, they kept the information confidential as it would be considered plagiarism. This study found no integration of ChatGPT from Khulna University in terms of student usage.

The study highlights significant limitations of ChatGPT, such as generating false references, outdated information, and biased responses. These issues stem from the model’s design, which relies on probabilistic predictions rather than factual verification. The “hallucination” of fake references, for instance, is a byproduct of the model’s lossy compression and inability to verify the accuracy of its outputs [75,76]. This limitation raises concerns about the reliability of AI-generated content for academic purposes and aligns with broader debates in the literature about the risks of over-reliance on generative AI in education [10,28,77]. Additionally, biases in ChatGPT’s training data may perpetuate stereotypes or cultural prejudices, which could negatively impact equitable learning environments [27,78].

### Contextualizing findings within theory

In the context of UGT, university students used ChatGPT as a search engine to satisfy their cognitive gratifications [28,59]. Some of them sought information and knowledge on various topics. Only a few of the students used the ChatGPT to satisfy their affective gratifications by seeking emotional experiences, such as humor, excitement, and relaxation from the ChatGPT’s responses to random questions [58]. Moreover, some students used ChatGPT to satisfy their integrative gratifications [57] by seeking self-awareness, self-esteem, and personal identity through ChatGPT’s suggestions on career planning or movie watchlists. Finally, some students used ChatGPT to satisfy their social integrative gratifications by seeking social interaction and companionship through using ChatGPT so that they can talk about it in their friend circle or suggest others to ease it and make their life easier [60]. It is essential to keep in mind that excessive reliance on ChatGPT can result in a reduction in one’s capacity for analytical thinking, attention span, and ability to solve problems. Although OpenAI’s GPT-4o is designed to guide learners through complex problems without providing direct answers—potentially fostering critical thinking and autonomy [20,21]—our findings reveal a different reality. Most students do not view ChatGPT as a tool to enhance critical thinking and show little concern for developing such skills. Instead, they primarily use AI for task completion and assistance, indicating a disconnect between the tool’s intended pedagogical role and actual usage.

### Implications for academic integrity

The misuse of ChatGPT for plagiarism and unethical practices poses challenges to academic integrity. Students may exploit its capabilities to bypass traditional plagiarism detection systems or generate assignments with minimal effort, undermining critical thinking and originality [10,77,79,80]. This trend reflects a broader need for educational institutions to adapt their policies and practices to address AI misuse. Without clear guidelines, the credibility of academic achievements could be compromised. In response to the rise of generative AI, universities like Flinders and Adelaide have introduced policies allowing limited AI use in academic work, provided students adhere to guidelines and demonstrate critical thinking alongside AI outputs [33]. Similarly, the University of Queensland advises students to consult course requirements before integrating AI into assessments [34]. Meanwhile, experimental studies caution that overreliance on tools like ChatGPT can diminish creativity and originality, particularly in creative fields, reinforcing the need for careful policy design and responsible use [54].

### Recommendations for educators

Based on the study’s findings, several actionable recommendations can enhance the responsible integration of ChatGPT in educational settings. Educators should integrate comprehensive AI literacy programs by developing structured modules within curricula that educate students about the strengths, limitations, and ethical use of AI tools. These modules need to specifically cover topics such as verification of AI-generated information, recognizing biased content, and understanding the implications of plagiarism and academic dishonesty.

Additionally, educators are encouraged to design assignments that are AI-proof by requiring personalized critical analysis, reflection, or creative synthesis that AI cannot easily replicate. Examples include critically evaluating ChatGPT-generated content or incorporating experiential learning and reflective practices.

Institutions should establish clear policies outlining acceptable and unacceptable uses of AI in coursework, explicitly defining plagiarism, ethical considerations, and proper citation practices. These guidelines must be communicated clearly and reinforced periodically.

Furthermore, institutions should conduct regular training sessions for faculty to enhance their understanding of AI capabilities and limitations, equip them with strategies to detect misuse, and leverage AI effectively to promote critical thinking and originality.

Robust assessment strategies that emphasize processes rather than merely outputs are essential. Implementing reflective journals, oral presentations, or peer-reviewed projects can ensure assessments genuinely reflect students’ intellectual engagement and personal efforts.

Finally, educators should encourage ethical dialogue and awareness by fostering open classroom discussions about ethical dilemmas and responsible AI use. This practice can facilitate students’ deeper understanding and internalization of academic integrity values by sharing experiences and challenges related to AI usage.

## Conclusion

This study sheds light on the multifaceted motivations driving university students’ engagement with ChatGPT, highlighting both its academic and non-academic applications. Despite the tool’s perceived efficiency in enhancing productivity and facilitating various tasks, the findings emphasize critical ethical concerns, particularly relating to academic integrity and the potential weakening of critical thinking skills.

Notably, the study has significant limitations. Primarily, the sample size of 20 participants from a single university in Bangladesh limits the generalizability of the findings. Given the global and diverse nature of ChatGPT usage, the narrowly defined geographical and cultural context restricts the applicability of the results to broader, international student populations. Furthermore, the small sample heightens the possibility of skewed perspectives, influenced by local educational practices, technological accessibility, and socioeconomic conditions.

Moreover, this research captures self-reported perspectives and behavioral intentions, which might differ significantly from actual behaviors. Students’ claimed intentions and experiences with ChatGPT could potentially differ from observed usage patterns. Thus, future research should expand this qualitative exploration to larger, more diverse populations, using mixed-method approaches that incorporate observational or experimental methodologies to validate reported behaviors against actual usage.

Future studies should also consider longitudinal designs to track changes in student interactions with AI over time, especially in response to technological advancements and evolving educational policies. Given these limitations, future research should utilize this study primarily as a methodological starting point, emphasizing the importance of rigorous qualitative techniques to explore complex phenomena like AI adoption in education, rather than drawing definitive conclusions from the specific findings presented here.

Overall, this research advocates for a balanced and ethically-informed integration of ChatGPT in educational practices. By proactively addressing limitations, clearly defining usage guidelines, and focusing on AI literacy, educational institutions can effectively harness AI’s potential while safeguarding academic standards and fostering robust critical thinking skills among students.

## Supporting information

S1 File Interview Questionnaire. (DOCX)

S2 File Inclusivity in Global Research. (DOCX)
