# Correction: How University students in Bangladesh engage with ChatGPT: A qualitative study

**DOI:** 10.1371/journal.pone.0344589

**Published:** 2026-03-09

**Authors:** Mir Hasib, Md. Shariful Islam

There is an error in affiliation 2 for author Md. Shariful Islam. The correct affiliation 2 is: Mass Communication and Journalism Discipline, Social Science School, Khulna University, Khulna, Bangladesh.
