# Supplementary material for: How University students in Bangladesh engage with ChatGPT: A qualitative study
Source: PLoS One. 2025 Sep 23;20(9):e0333089. doi: 10.1371/journal.pone.0333089 (PMC12456799; doi:10.1371/journal.pone.0333089)
Supplement: S1 File — (DOCX) [file pone.0333089.s001.docx]

# **Supporting information**

## **S2 File. Interview Questionnaire**

(Translated from Bangla to English)

***Research Description (verbal)***

***Consent (verbal)***

**Section 1: Participant Background and Initial Perceptions**

Can you provide your name, age, gender, academic discipline, and year of study?

How did you first learn about ChatGPT, and what motivated you to start using it? *(If never used ChatGPT, conclude the interview)*

Did you have any initial hesitations or concerns before using ChatGPT? If so, how did your perception change over time?

**Section 2: Academic Use of ChatGPT**

How do you use ChatGPT for academic tasks (e.g., assignments, research, presentations, exam preparation)?

Can you share a specific example of how ChatGPT helped you in an assignment or project?

How does ChatGPT compare to traditional study resources like books, journal articles, or search engines?

Have you ever used ChatGPT as an alternative to taking lecture notes? Why or why not?

Have you discussed ChatGPT use with professors? If so, what was their reaction?

**Section 3: Writing Assistance and Research Accuracy**

How do you use ChatGPT for writing tasks (e.g., grammar checking, paraphrasing, expanding content)?

Have you encountered any issues with ChatGPT-generated references or citations? How do you verify its accuracy?

Have you ever received feedback from an instructor regarding work where you used ChatGPT? What was their response?

Do you believe using ChatGPT has improved your writing or analytical skills? Why or why not?

**Section 4: Ethical Considerations and Academic Integrity**

Do you consider the use of ChatGPT in academic work ethical? Why or why not?

Have you or your peers used ChatGPT to bypass plagiarism detection? If yes, how?

Have you used ChatGPT in an exam setting or for graded assignments? If so, what was your rationale?

Should universities introduce training or guidelines on responsible ChatGPT use? What should be included?

**Section 5: Non-Academic and Creative Uses**

Besides academic work, how do you use ChatGPT? (e.g., job applications, content creation, personal decision-making)

Have you experimented with ChatGPT for creative projects (e.g., storytelling, poetry, brainstorming ideas)?

Do you use ChatGPT for entertainment, humor, or casual conversations? Can you share an interesting or humorous interaction?

Have you used ChatGPT in collaborative work or group projects? If so, how did it help?

Section 6: ChatGPT vs. Traditional Search Engines (5 mins)

Have you replaced Google or other search engines with ChatGPT? Why or why not?

What are the biggest advantages and disadvantages of using ChatGPT instead of traditional search engines?

**Section 7: Future Expectations and Long-Term Impact**

How has ChatGPT impacted your learning experience and productivity?

Do you feel that ChatGPT understands your academic needs? What improvements would you like to see?

How do you see yourself using ChatGPT in the next five years?

Do you think ChatGPT will play a larger role in education in the future? If so, how?

Would you recommend ChatGPT to other students? Why or why not?

***Conclude and appreciate for participation.***
